# Consolidation of Medical Colleges

**Published:** 1898-08

**Authors:** 


### CONSOLIDATION OF MEDICAL COLLEGES.

This seems a year of consolidation, certainly as far as medical schools are concerned. In the last issue of THE JOURNAL we spoke of the union of the Atlanta and Southern Medical Colleges, of this city, forming what is now known as the Atlanta College of Physicians and Surgeons. News comes to us of still further unions in this direction.

The Niagara University Medical College and the Buffalo University Medical School have consolidated under the Medical Department of the University of Baltimore.

The Cornell University, of Ithaca, N. Y., has also embraced unto itself a medical department by reorganizing and taking most of the men from the old New York Medical College.

The University of the City of New York has appropriated the old Bellevue Hospital Medical College, and henceforth it will be a department of that institution.

We cannot but say that we are glad to see these changes. In two instances two medical institutions have been made one, thus reducing the number of medical colleges in the United States. The number of medical colleges in the United States is alarming, and it will soon be so, with the present rate of increase, that each State can claim the existence of four or five medical colleges within its borders. Already some States, and even cities, are far in excess of this. With this state of affairs confronting us, we can but hold a pessimistic view of the future medical profession in this country. Medical colleges are beginning to be nothing more than money-making machines, either by money received from students who ought to be in a second-grade country school, or by being able to prefix to their names the word professor they are better enabled to deceive the public as to their ability. This is no overdrawn picture. Then, again, the polyclinic schools are doing harm, as Dr. Scheppegell, of New Orleans, has shown in a recent paper, by giving students certificates, after an attendance of six weeks, allowing them to branch out as "full-fledged specialists," thus bringing the world of specialism into still more disrepute. The field of medicine certainly does not offer an inviting field for young men of brains. "But the end is not yet."

---

OUR colaborer and editor, Dr. Luther B. Grandy, has entered the volunteer service of the United States Army. He has been appointed chief surgeon, with the rank of major, in the Third Regiment of Georgia Volunteers, which is now in camp at Griffin, Ga. While we miss his active labors on THE JOURNAL, yet we expect to hear from him frequently, giving to the readers of THE JOURNAL some medical items with warlike tinge. Our best wishes go with him in his new duties.

